# Tourniquet Test for Dengue Diagnosis: Systematic Review and Meta-analysis of Diagnostic Test Accuracy

**DOI:** 10.1371/journal.pntd.0004888

**Published:** 2016-08-03

**Authors:** Antonio Jose Grande, Hamish Reid, Emma Thomas, Charlie Foster, Thomas C. Darton

**Affiliations:** 1 Universidade do Extremo Sul Catarinense, Criciuma, SC, Brazil; 2 Nuffield Department of Population Health, University of Oxford, Oxford, United Kingdom; 3 British Heart Foundation Centre on Population Approaches for Non-Communicable Disease Prevention, Nuffield Department of Population Health, University of Oxford, Oxford, United Kingdom; 4 Department of Infection, Immunity and Cardiovascular Disease, University of Sheffield, Sheffield, United Kingdom; 5 Oxford University Clinical Research Unit, Hospital for Tropical Diseases, Ho Chi Minh City, Vietnam; Santa Fe Institute, UNITED STATES

## Abstract

**Background:**

Dengue fever is a ubiquitous arboviral infection in tropical and sub-tropical regions, whose incidence has increased over recent decades. In the absence of a rapid point of care test, the clinical diagnosis of dengue is complex. The World Health Organisation has outlined diagnostic criteria for making the diagnosis of dengue infection, which includes the use of the tourniquet test (TT).

**Purpose:**

To assess the quality of the evidence supporting the use of the TT and perform a diagnostic accuracy meta-analysis comparing the TT to antibody response measured by ELISA.

**Data Sources:**

A comprehensive literature search was conducted in the following databases to April, 2016: MEDLINE (PubMed), EMBASE, Cochrane Central Register of Controlled Trials, BIOSIS, Web of Science, SCOPUS.

**Study Selection:**

Studies comparing the diagnostic accuracy of the tourniquet test with ELISA for the diagnosis of dengue were included.

**Data Extraction:**

Two independent authors extracted data using a standardized form.

**Data Synthesis:**

A total of 16 studies with 28,739 participants were included in the meta-analysis. Pooled sensitivity for dengue diagnosis by TT was 58% (95% Confidence Interval (CI), 43%-71%) and the specificity was 71% (95% CI, 60%-80%). In the subgroup analysis sensitivity for non-severe dengue diagnosis was 55% (95% CI, 52%-59%) and the specificity was 63% (95% CI, 60%-66%), whilst sensitivity for dengue hemorrhagic fever diagnosis was 62% (95% CI, 53%-71%) and the specificity was 60% (95% CI, 48%-70%). Receiver-operator characteristics demonstrated a test accuracy (AUC) of 0.70 (95% CI, 0.66–0.74).

**Conclusion:**

The tourniquet test is widely used in resource poor settings despite currently available evidence demonstrating only a marginal benefit in making a diagnosis of dengue infection alone.

**Registration:**

The protocol for this systematic review was registered at PROSPERO: CRD42015020323.

## Author Summary

Dengue is an infectious disease transmitted by mosquitoes in the Tropics. There are 2.5 billion people around the world at risk. Dengue presents as an acute febrile illness with symptoms including headache, bone or joint and muscular pains and rash. The objective of this study is to perform a diagnostic accuracy meta-analysis comparing the use of the Tourniquet Test (TT) to a laboratory assay standard (ELISA) for making a diagnosis of dengue infection. A comprehensive literature search (to April, 2016) was conducted to map and assess the quality of the available evidence, using the following databases: MEDLINE (PubMed), EMBASE, Cochrane Central Register of Controlled Trials, BIOSIS, Web of Science, SCOPUS. We included 16 studies with 28,739 participants in the meta-analysis. Pooled sensitivity for dengue diagnosis by TT was 58% (95% Confidence Interval (CI), 43%-71%) and the specificity was 71% (95% CI, 60%-80%). In the pooled subgroup analysis sensitivity for dengue fever diagnosis was 55% (95% CI, 52%-59%) and the specificity was 63% (95% CI, 60%-66%). The tourniquet test is widely used in resource poor settings despite currently available evidence demonstrating only a marginal benefit in making a diagnosis of dengue infection alone.

## Introduction

Dengue is an arboviral infection ubiquitous to tropical and sub-tropical regions,[1–3] where it is transmitted by domesticated day-biting mosquitoes including *Aedes aegypti*. After an incubation period of 4–10 days (mean, 7 days), illness onset is abrupt (with headache, fever, myalgia/arthralgia and rash) and can last up to 14 days[4–8]. Four virus serotypes are in circulation around the world (DEN-1, DEN-2, DEN-3, DEN-4)[9,10], with specific “Asian” genotypes within serotypes DEN-2 and DEN-3 being associated with severe dengue infection particularly in secondary infections [11,12]. According to WHO estimates 50–100 million new dengue infections occur annually, resulting in 500,000 cases of DHF and 22,000 deaths[11,12]. It is thought that approximately 2.5 billion people, or 40% of the world’s population are at risk of dengue infection, with important factors including warm and humid climate, overcrowding and residence in major urban centers[11–14].

Virus transmission can cause a spectrum of illness from subclinical to severe dengue infection characterized by plasma leakage, haemorrhage and end-organ impairment. Characterisation of specific phenotypes of infection is complex and has recently changed[11–14]. Clinically, dengue fever presents as an acute febrile disease with symptoms of headache, bone or joint and muscular pains, rash and leukopenia. Traditionally a further two stages were described, consisting of dengue haemorrhagic fever (DHF), characterized by high fever, haemorrhagic phenomena, often with hepatomegaly. In severe cases, further signs of circulatory failure may develop culminating in dengue shock syndrome (DSS), which is associated with poor outcomes. More recent consensus guidance[12] recommends distinction of dengue illness into dengue (with or without warning signs which may precede the development of more severe infection) and severe dengue (encompassing the manifestations of severe plasma leakage, severe bleeding or severe end-organ involvement)[11,12].

The clinical diagnosis of dengue is challenging as the symptoms are non-specific and common to many other infections[10–12], notably malaria and other arboviral infections. To aid diagnosis, specifically during the initial, acute, febrile phase which may last 2–7 days after the development of fever, the WHO recommend the use of the Tourniquet Test (TT, also known as the Rumpel-Leede or Hess test) to support diagnostic decision-making [13,15–21]. As an inexpensive, quick and easy to perform procedure, use of the TT has become widespread in clinical practice globally. The TT is a marker of capillary fragility and can be undertaken by inflating a blood pressure cuff around the upper arm to the point midway between the individual’s systolic and diastolic blood pressures and leaving it inflated for 5 minutes. The cuff is subsequently released and after two minutes the number of petechiae below antecubital fossa are counted. The test is positive if more than 10 petechiae are present within a square inch of skin on the arm[11,12]. The clinical diagnosis of dengue may be confirmed by laboratory testing, which in many settings involves the measurement of an antibody response (IgM or IgG) by ELISA[3], for years considered to be the diagnostic standard[22]. This test is less sensitive in the first 5 days after exposure and frequently relies on testing of paired sera samples. Newer tests available in some centres include reverse-transcriptase PCR (polymerase chain reaction) or direct antigen detection (non-structural protein 1). While these tests are likely to offer an improvement in diagnostic accuracy, the cost and current limitation of not detecting all serotypes limits their application.

The evidence to support the recommendation and widespread use of the TT to aid the diagnosis of dengue fever is mixed with variable sensitivity and specificity being reported previously(15–21). The aim of this study is to map the evidence, assess the quality of the studies and perform a diagnostic accuracy meta-analysis of the diagnosis of dengue using the TT compared to ELISA.

## Methods

### Data Sources and Searches

The protocol for this systematic review was registered at PROSPERO (International prospective register of systemic reviews, http://www.crd.york.ac.uk/PROSPERO/display_record.asp?ID=CRD42015020323). We searched Medical Literature databases Analysis and Retrieval System Online (Medline), Excerpta Medical Database (EMBASE), Allied and Complementary Medicine Database (AMED), Global health, Biological Abstracts/Reports, Reviews, Meetings (BIOSIS) altogether through OVID. Latin American and Caribbean Health Sciences (LILACS) and the Cochrane Library through their website for relevant publications until April 2016. Additionally, we searched the WHO ICTRP (International Clinical Trials Registry Platform) and ClinicalTrials.gov for completed and ongoing studies.

The search, performed according to the Cochrane Highly Sensitive Search Strategy, used the following terms: “Rumpel-Leede” OR capillary OR “blood pressure cuff” OR petechiae OR tourniquet OR “Hess” AND dengue. The search was sensitive, we used no study filters and no language or publication restrictions. We checked the reference lists of all primary studies included for additional references. There were no language or publication restrictions on our search.

We included cross-sectional and cohort studies that evaluated the diagnostic accuracy of tourniquet test for dengue infection. Both retrospective and prospective studies that consecutively or randomly selected patients were included, together with studies that used delayed verification for gold standard. We included studies looking at patients presenting with fever who were subsequently tested for dengue using both the TT (index test) and ELISA detection of antibody response (reference standard).

For this review, definitions of dengue were used according to those proposed by the WHO[11,12], as these were the definitions used during the time period from which studies were drawn. For the purposes of this meta-analysis, ‘dengue’ was considered to consist of non-severe ‘dengue fever’ and ‘haemorrhagic dengue fever’, defined as follows. Dengue fever included fever plus 2 or more symptoms of nausea/vomiting, rash, aches and pains. Dengue hemorrhagic fever (DHF) was considered as infection accompanied by haemorrhagic manifestations such as petechiae and mucosal or gastro-intestinal bleeding[11,12].

Three comparisons were performed; TT vs. ELISA to diagnose dengue (i.e. both non-severe dengue fever plus DHF; TT vs. ELISA to diagnose dengue fever and TT vs. ELISA to diagnose DHF.

### Study Selection

Two review authors (AJG, HR) independently assessed all studies identified from the database searches by screening titles and abstracts using the Review Management website Covidence (http://www.covidence.org). We separated potential studies for full-text reading. A third review author (ET) resolved any disagreements, and reasons for including and excluding trials were recorded.

### Data Extraction and Quality Assessment

Two review authors (AJG, HR) independently extracted data from the included studies using a standard data extraction form. With this form we extracted information of study design, participant description, index test description, reference test description, dengue classification and total number of participants. A 2x2 table was created for each study comparing both tests.

All included studies were assessed for their methodological quality using the quality assessment tool for diagnostic accuracy studies (QUADAS-2)[23]. The tool is composed of 17 items regarding study patient selection, index test, reference standard and flow and timing. For each domain mentioned there are items for risk of bias and applicability. Items were scored as positive (low risk of bias), negative (high risk of bias), or insufficient information (unclear). A description of each assessment was described in the results section.

### Data Synthesis and Statistical Analysis

For each study, a 2x2 contingency table was constructed. We calculated sensitivity, specificity and likelihood ratios (LRs). When the primary study had 0 in a cell of the 2x2 table, the value of 1 was added, so calculations could be done[24], this only happened in one study(17). We planned to exclude primary studies reporting two cells with 0, but this did not occur.

The sensitivity, specificity and LRs were pooled from each study and a forest plot was generated with 95% confidence intervals. Due to the variability in diagnostic data, we logit-transformed sensitivity and specificity for each primary study and for the aggregate result, considering variability within-study and between-study. The output results are random effects estimates of the mean sensitivity and specificity with corresponding 95% CI. The weighing considered the inverse of the standard error, so indirectly to the sample size reported in the studies. Inconsistency (I^2^) was explored as an indicator of statistical heterogeneity[24]. Summary receiver operating characteristic (ROC) curves were generated with calculation of area under the curve (AUC) as an indicator of test accuracy. To assess for the possibility of publication bias, we constructed funnel plots to visually assess for signs of asymmetry [25].

Statistical analyses were performed with Stata v10.0 (StataCorp LP, Texas, USA) and with RevMan v5.3 (The Nordic Cochrane Centre, Copenhagen, Denmark)[26].

## Results

**Fig 1:**
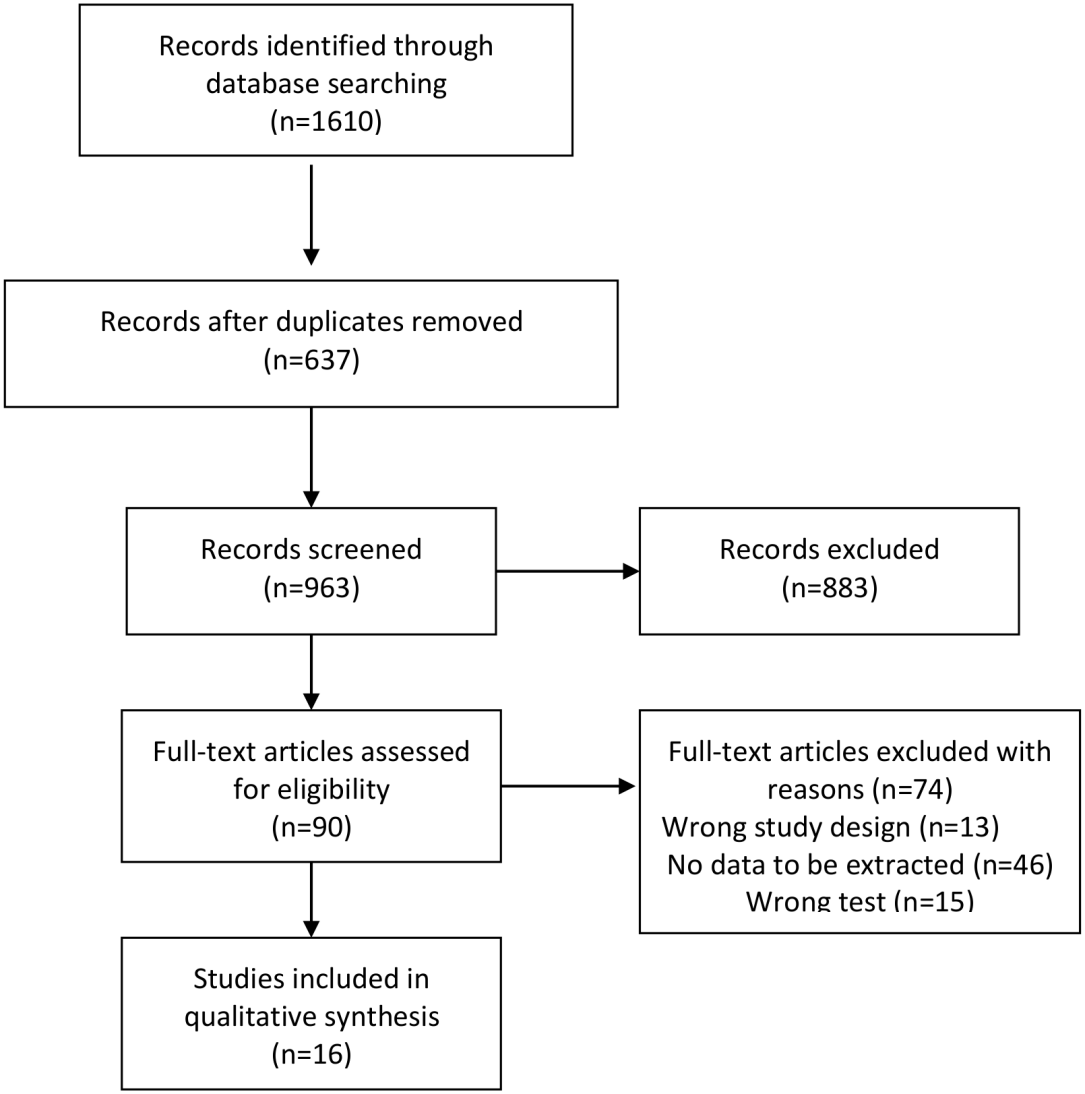
Flow chart of systematic review process.

We identified 1610 studies of which 637 were excluded as duplicates (Fig 1). A total of 973 studies were assessed on the basis of the title and abstract, of which 883 were excluded because they did not fulfill inclusion criteria. Full text studies were retrieved for 90 titles, of which 74 were excluded (Table 1): unable to extract absolute numbers of true positives, false positives, false negatives, and true negatives (n = 46), wrong test (n = 15) and wrong study designs (n = 13)[4–8,27–95].

**Table 1:** Excluded studies with reasons.

Study	Reason for exclusion	Study	Reason for exclusion	Study	Reason for exclusion
**Ahmed 2001**[27]	No data to be extracted	**Gregory 2010**[96]	No data to be extracted	**Muhammad 2006**[7]	No data to be extracted
**Arif 2009**[28]	No data to be extracted	**Guzman 1996**[46]	No data to be extracted	**Munir 1982**[8]	No data to be extracted
**Awasthi 2012**[29]	Wrong test	**Hanif 2011**[47]	No data to be extracted	**Munir 2014**[60]	No data to be extracted
**Ayyub 2006**[30]	No data to be extracted	**Horstick 2012**[48]	Wrong study design	**Namvongsa 2013**[61]	No data to be extracted
**Bandyopadhyay 2006**[31]	Wrong study design	**Horstick 2014**[49]	Wrong study design	**Narayanan 2003**[62]	No data to be extracted
**Cavalcanti 2010**[32]	Wrong test	**Ismail 2006**[50]	No data to be extracted	**Naskar 2014**[63]	Wrong test
**Chairulfatah 1995**[33]	No data to be extracted	**Jhamb 2010**[51]	Wrong study design	**Nunes Araujo 2003**[64]	Wrong test
**Chuansumrit 2010**[34]	No data to be extracted	**Kabra 1999**[4]	No data to be extracted	**Padbidri 1995**[65]	Wrong study design
**Chutinimitkul 2005**[35]	Wrong test	**Kalayanarooj 2003**[52]	Wrong study design	**Pancharoen 2001**[66]	No data to be extracted
**Daniel 2005**[36]	No data to be extracted	**Kapse 2003**[53]	Wrong study design	**Pandey 2008**[67]	Wrong test
**Diaz Quijano 2008**[37]	No data to be extracted	**Kittitrakul 2015**[54]	No data to be extracted	**Phuong 2006**[68]	No data to be extracted
**Diaz Quijano 2009**[38]	Wrong test	**Kulkarni 2010**[55]	No data to be extracted	**Prathyusha 2013**[69]	No data to be extracted
**Diaz Quijano 2010**[39]	No data to be extracted	**Mamani 2010**[56]	No data to be extracted	**Quiroz Moreno 2006**[70]	Wrong test
**Eram 1979**[40]	No data to be extracted	**Manjith 2002**[5]	No data to be extracted	**Ramirez Ronda 1987**[71]	Wrong study design
**Faridi 2008**[41]	No data to be extracted	**Meltzer 2012**[57]	Wrong test	**Ramirez Zepeda 2009**[72]	No data to be extracted
**Filho 2012**[42]	No data to be extracted	**Mendez 2003**[6]	No data to be extracted	**Rao 2013**[73]	No data to be extracted
**Fonseca 2012**[43]	Wrong test	**Mishra 2014**[58]	Wrong test	**Restrepo 2012**[74]	Wrong test
**Gomber 2001**[44]	No data to be extracted	**Mourao 2007**[59]	No data to be extracted	**Ribeiro 2010**[75]	No data to be extracted
**Richards 1997**[76]	No data to be extracted	**Sri 1999**[82]	Wrong test	**Villar 2011**[88]	Wrong test
**Rigau Perez 1997**[77]	No data to be extracted	**Sunil 2001**[83]	No data to be extracted	**Wali 1999**[89]	No data to be extracted
**Setiati 2007**[78]	No data to be extracted	**Tham 1996**[84]	Wrong study design	**Whitehorn 2010**[90]	Wrong study design
**Shah 2006**[79]	No data to be extracted	**Tomashek 2010**[85]	No data to be extracted	**Yadav 2008**[92]	Wrong study design
**Sharmin 2013**[80]	No data to be extracted	**Uddin 2014**[86]	No data to be extracted	**Yewale 2009**[93]	Wrong study design
**Wiwanitkit 2005**[91]	Wrong test	**Zhang 2005**[95]	No data to be extracted	**Zhang 2007**[94]	No data to be extracted
**Shibani 2006**[81]	Wrong study design	**Vargas 2001**[87]	No data to be extracted		

The remaining 16 studies were included in the systematic review and meta-analysis[13–21,96–102] (Table 2), 14 of which were prospective cohorts while two were retrospective cohort studies. The number of participants in each study ranged from 79 to 13,548. Ten studies were from countries in Asia and six studies from Latin America.

**Table 2:** Characteristics of included studies.

Study/ Country	Design	Participants description	Index test description	Reference test description	Dengue classification	Total number of participants	TP	FP	FN	TN
Antunes 2013[13]/Brazil	Retrospective cohort study	All reported cases of suspected dengue in Belo Horizonte between 2001 and 2006. Not reported who the participants were. Children and adults mixed	No mention about when TT was done. ≥20 petechiae per one square inch. ≥10 petechiae per one square inch children	ELISA IgM-anti-DENV test. Not reported when blood collection was performed.	Classic dengue, Dengue hemorrhagic fever, Complicated dengue	9,836 cases of suspected dengue infection	774	789	3273	5000
Diaz 2006[15]/ Colombia	Prospective cohort study	The population consisted of patients ≥ 12 years who went to health centers of the metropolitan area of Bucaramanga, Colombia, during the period April 2003 to April 2004.	TT was performed in the first clinical evaluation. Cut-off ≥20 petechiae per one square inch	IgM antibody capture enzyme-linked immunosorbent assay (MAC-ELISA IgM) between 48–96 hours of first physical manifestations	Dengue and acute febrile illness	262 Patients with acute febrile illness	54	41	71	53
Falconar 2012[14]/Colombia	Prospective cohort study	100 patients aged 1–27 years old who presented at the clinics. This study was performed during a 2007 epidemic of severe dengue disease	No mention about when TT was done. Cut-off ≥20 petechiae per one square inch	ELISA IgM-anti-DENV test. First blood samples on either the day of onset of febrile illness (day 0), or day 1 to 3 (< 72 hours) after the onset of febrile illness	Dengue hemorrhagic fever and dengue shock syndrome	100 patients	13	14	20	53
Gregory 2011[96]/Porto Rico	Prospective cohort study	The surveillance period was from September 29, 2009 through December 18, 2009. All patients presenting for medical care at the ED of Saint Luke’s Episcopal Hospital who met the case definition above were enrolled in the surveillance system. Fever that persisted for 2 to 7 days without identified source. The participants age were mixed <15 years and age>15 years.	TT was conducted during the duration of fever. Cut-off ≥10 petechiae per one square inch	ELISA Immuno DOT kit. Blood tests were conducted at the discretion of the attending physician.	Dengue	284 patients with acute febrile illness	16	38	15	178
Halsey 2013[16]/ Peru	Prospective cohort study	Participants aged 3–18 years old, clinic-based passive febrile surveillance component and a community-based active febrile surveillance component. during September 2006–March 2011 first cohort. April 2008–2011 second cohort	TT conducted at day 0 and day 7. Cut-off ≥20 petechiae per one square inch	ELISA for DENV IgM. Day 0, day 7, day 14 and day 21.	Dengue	13,548 persons with febrile disease	3078	2632	2464	5374
Ho 2013a[17]/ Taiwan	Retrospective cohort study	suspected dengue cases consist of 100 children (≤ 18 years) and 481 adults. hospital-based study was conducted at National Cheng Kung University Hospital from Jan. to Dec., 2007. patients with reported or documented fever of ≥38°C of less than 7 days’ duration and two or more symptoms or signs	Not clear when TT was conducted. Cut-off ≥20 petechiae per one square inch	ELISA for DENV IgM. Not clear when blood test was conducted.	Dengue, dengue hemorrhagic fever or dengue shock syndrome	100 children and 481 adults	178	1	85	68
Hoang 2006[18]/ Viet nam	Prospective cohort study	From April 2001 to April 2002, 2108 children and adults were enrolled in this study. twelve community health posts and one clinic at the provincial malaria station, Binh Thuan Province, Vietnam	TT was conducted in the first visit. No cut-off reported	IgG and IgM-Capture ELISA. Blood test conducted in day 0 and 3 weeks later for reassessment.	Dengue	2096 patients	23	38	211	425
Kalayanarooj 1997[20]/ Thailand	Prospective cohort study	Healthy children from 6 months to 15 years old who presented to Bangkok Children’s Hospital between 25 April and 14 December 1994 fever for .72 h, oral temperature ≥38.5 C recorded in the clinic	TT was performed each day until discharge. Cut-off ≥20 petechiae per one square inch	IgM and IgG ELISA. Day 0, and 7 or 8 days after discharge	Dengue, dengue fever, dengue hemorrhagic fever	172 children	33	42	18	66
Kalayanarooj 1999[19]/ Thailand	Prospective cohort study	Twelve febrile patients were enrolled each week between 1994 and 1997 from the outpatient department of two hospitals, Children’s Hospital in Bangkok and Kampangpet Provincial Hospital. The patients met the following criteria: age 6 months to 15 years, had temperature ³ 38.5°Celsius for < 72 hours, had facial flushing and no obvious source of infection	Physician did TT everyday. Cut-off ≥20 petechiae per one square inch	ELISA. Blood test was done every morning	Dengue fever, dengue hemorrhagic fever	649 febrile children	286	172	32	159
Kalayanarooj 2011[98]/ Thailand	Prospective cohort study	Healthy children from 6 months to 15 years old with suspected dengue who were admitted to the Dengue Unit, Queen Sirikit National Institute of Child Health between June-August 2009 presented with shock or had a history of high fever with bleeding symptoms	Not reported when TT was conducted. Cut-off ≥20 petechiae per one square inch	Dengue was determined by polymerase chain reaction (PCR) and/or by serology. Not clear when blood was collected	Dengue fever, dengue hemorrhagic fever	356 suspected dengue patients	191	14	83	10
Mayxay 2011[98]/ Lao	Prospective cohort study	Adult patients (aged >15 years) admitted with undifferentiated fever of <7 days with a clinical diagnosis of dengue infection by the admitting physicians were enrolled. The study was conducted between October 2006 and October 2007 at the Adult Infectious Disease Ward of Mahosot Hospital	Two physicians performed TT within 24 h of admission. Cut-off ≥20 petechiae per one square inch	ELISA at admission and 7–14 days after discharged	Dengue primary and secondary infection	277 patients	58	10	112	54
Mendez 2013[99]/ Colombia	Prospective cohort study	We performed a prospective collection of pediatric patients between 2–12 years of age with acute febrile syndrome of origin is not apparent, admitted between June 2007 and April 2008 in the Four emergency services institutions Bucaramanga, Colombia	TT was conducted everyday. Cut-off ≥20 petechiae per one square inch	MAC-ELISA. At the sixth day of admission blood test was done to confirm dengue	Dengue	129 participants	40	26	26	33
Norlijah 2006a[100]/ Malaysia	Retrospective cohort study	Healthy children of 6 months to 12 years admitted at children's hospital in Kuala Lumpur between June and August 2001.	TT was conducted at day 0. Cut-off ≥20 petechiae per one square inch. If test negative it would be repeated until discharge	Monoclonal antibody capture enzyme immunoassay conducted at admission.	Dengue	79 subjects	48	13	10	4
Phuong 2002[101]/ Viet nam	Prospective cohort study	Children aged between 1–15 years, hospitalized between June 1996 and June 1998 with a diagnosis of suspected dengue infection (history of fever <7 days)	A standard tourniquet test was first performed on the right arm by a nurse. A series of six sites on the flexor and extensor aspects of the forearm. Cut-off ≥20 petechiae per one square inch Not reported when TT was done	ELISA. Not reported when the blood collection was performed	Dengue fever, Dengue hemorrhagic fever	1136 children	248	46	350	261
Sawasdivorn 2001[21]/ Thailand	Prospective cohort study	Patients admitted with a provisional diagnosis of dengue infection or suspected dengue infection age groups ranging from 1–13 years. September 1998 and September 1999	TT was performed daily. No cut-off reported	ELISA conducted at admission and 10–14 days later	Dengue fever, Dengue hemorrhagic fever, dengue infection	176 patients	39	24	6	14
Sirivichayakul 2012[102]/ Thailand	Prospective cohort study	Healthy boys and girls aged 3–10 years at the time of recruitment, living in Muang District or nearby villages.	Not reported when TT was done. Cut-off ≥20 petechiae per one square inch	ELISA. Blood samples for dengue collected each 7 days	Dengue fever, Dengue hemorrhagic fever	259 patients	118	21	20	33

All the analysis showed high levels of heterogeneity, represented by an I^2^ ranging from 75% to 100%. Given this considerable heterogeneity between studies, we performed a random effects meta-analysis presented below.

### Methodological Quality of Included Studies

**Fig 2:**
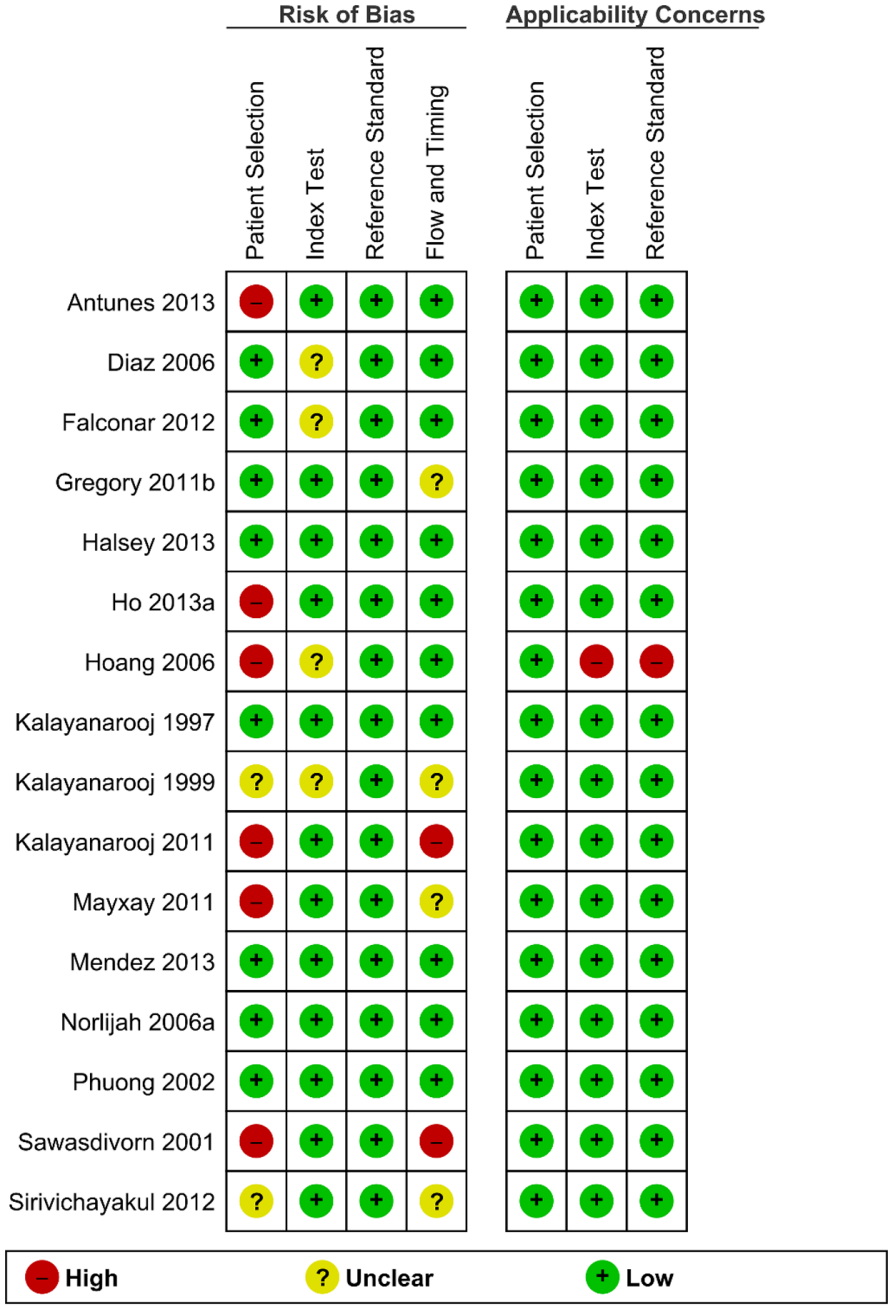
Quality Assessment of Diagnostic Accuracy Studies II.

We used the instrument QUADAS-2, which is composed of four quality categories (patient selection, reference standard, index test, and flow and timing), to critically appraise each included study (Fig 2). Six studies (33%) were considered to have high risk of bias in patient selection due to inclusion of patient data from a database, raising the possibility of bias from multiple assessors, or selection of patients with pre-existing disease. Two studies (17%) had not adequately described their sampling methods, so were classified as unclear risk. Eight studies (50%) were low risk of bias for patient selection.

Considering the Reference standard category (ELISA), all studies were considered low risk of bias. For the Index test category, four studies (25%) had not clearly described the process used to conduct the TT, blind assessors or train assessors.

For the flow and timing category, only two studies (12.5%) were considered at high risk of bias as the TT was repeated multiple times over a period of several days. Four studies (25%) were considered unclear risk due to lack of information of withdrawals and appropriate sequencing of tests.

### Dengue vs ELISA

**Fig 3:**
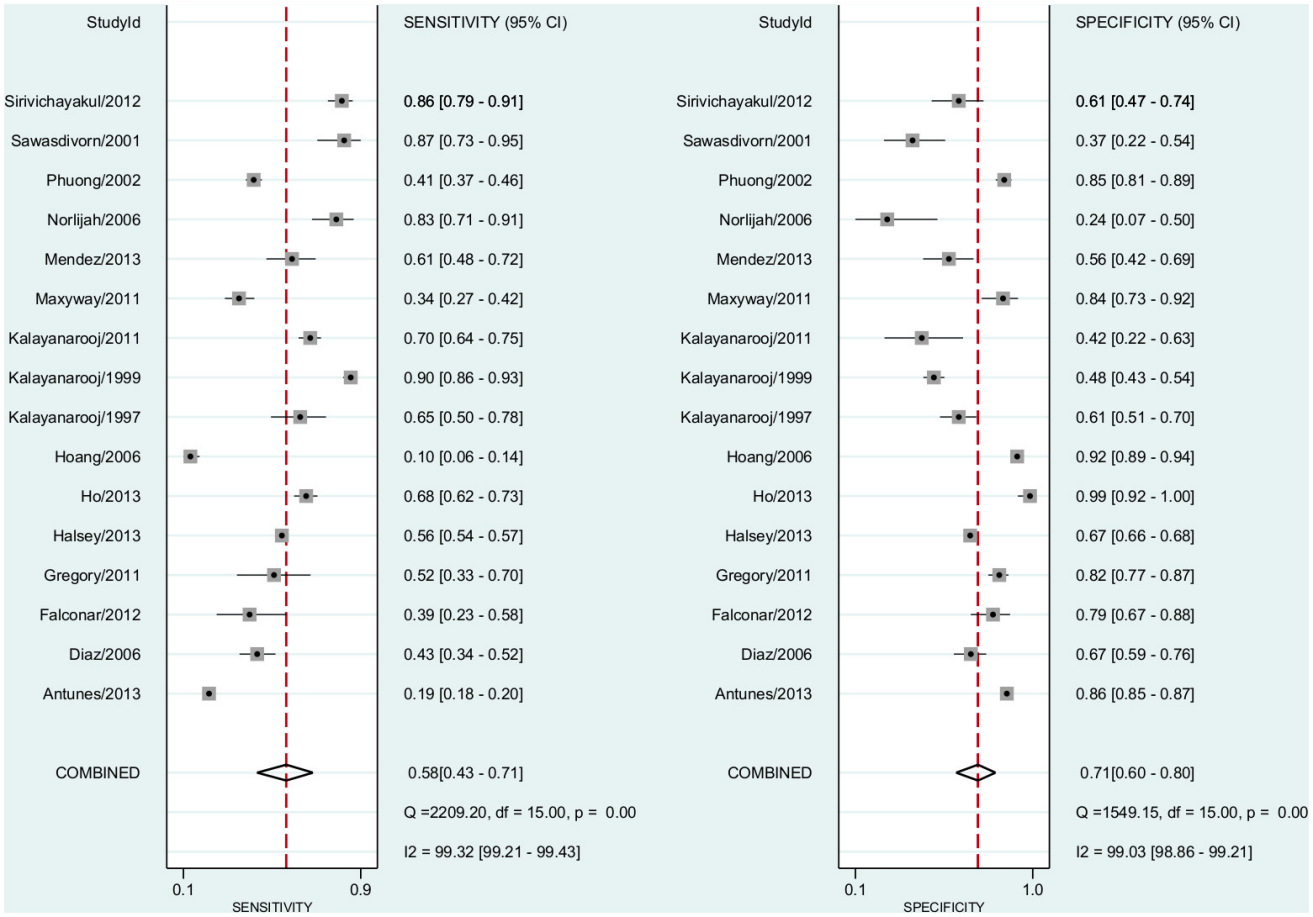
Forest plot for individual studies and pooled sensitivity and specificity for Dengue x ELISA. Q: chi-squared statistic; df: degrees of freedom; I^2^: inconsistency of studies’ results; Dashed line means the mean number found across studies.

**Fig 4:**
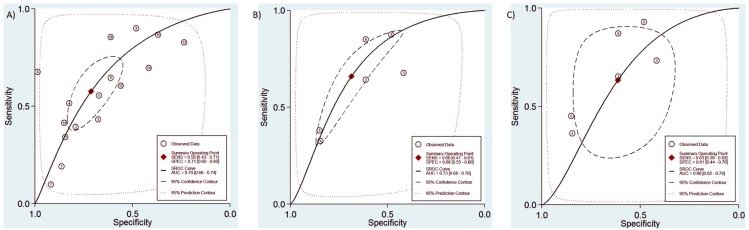
RoC curve for all three comparisons conducted in the study. A = Dengue x ELISA; B = Dengue fever x ELISA, C = Dengue haemorrhagic fever x ELISA.

In this comparison, we included all 16 studies including both non-severe dengue fever and DHF cases. The pooled sensitivity for dengue diagnosis was 0.58 (95% CI, 0.43–0.71) and the specificity was 0.71 (95% CI, 0.60–0.80)(Fig 3). The positive predictive value was 1.63 (95% CI, 1.45–1.82). The negative predictive value was 0.60 (95% CI, 0.51–0.71). The Diagnostic Odds Ratio was 2.88 (95% CI, 2.17–3.83). The area under the curve was 0.70 (95% CI 0.66–0.74)(Fig 4A).

### Dengue Fever vs ELISA

**Fig 5:**
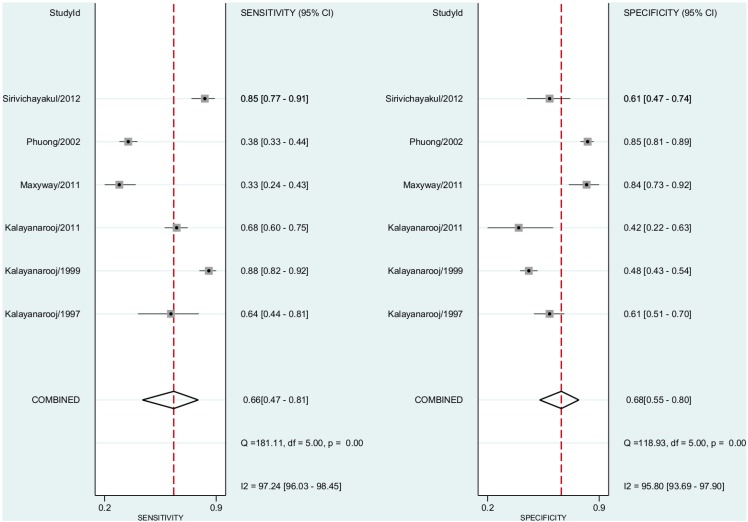
Forest plot for individual studies and pooled sensitivity and specificity for Dengue Fever x ELISA. Q: chi-squared statistic; df: degrees of freedom; I2: inconsistency of studies’ results; Dashed line means the mean number found across studies.

In this comparison, we included six studies. The pooled subgroup analysis sensitivity for dengue fever diagnosis was 0.66% (95% CI, 0.47–0.81) and the specificity was 0.68 (95% CI, 0.55–0.80)(Fig 5). The positive predictive value was 1.81 (95% CI, 1.45–2.25). The negative predictive value was 0.52 (95% CI, 0.36–0.75). The Diagnostic Odds Ratio was 3.80 (95% CI, 2.40–6.00). The area under the curve was 0.73 (95% CI 0.68–0.76)(Fig 4B).

### Dengue Haemorrhagic Fever vs ELISA

**Fig 6:**
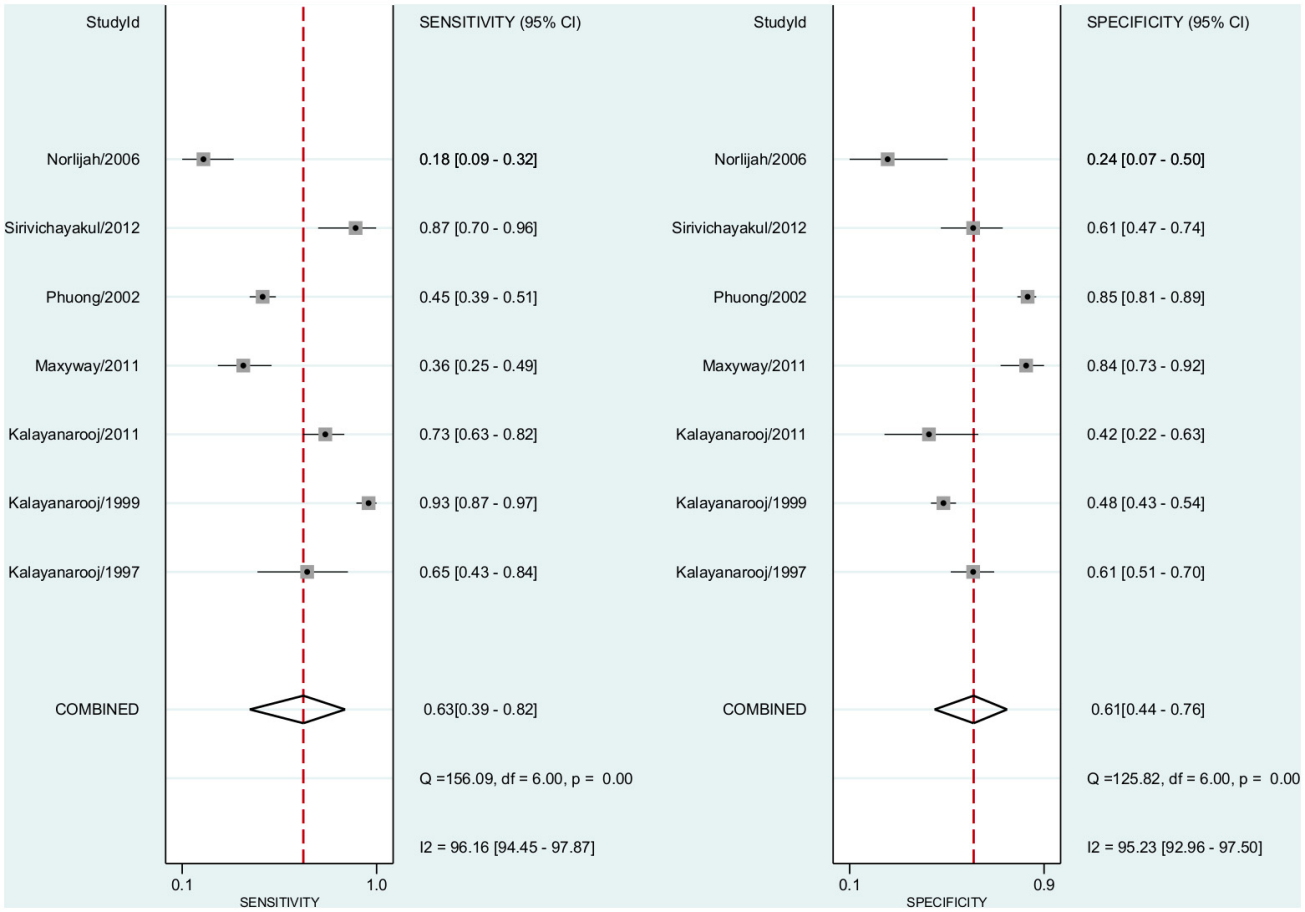
Forest plot for individual studies and pooled sensitivity and specificity for Dengue Haemorragic Fever x ELISA. Q: chi-squared statistic; df: degrees of freedom; I2: inconsistency of studies’ results; Dashed line means the mean number found across studies.

In this comparison, we included seven studies. In the pooled subgroup analysis, sensitivity for dengue haemorrhagic fever diagnosis was 0.63 (95% CI, 0.39–0.82) and the specificity was 0.60 (95% CI, 0.48–0.70). The positive predictive value was 1.54 (95% CI, 1.06–2.24)(Fig 6). The negative predictive value was 0.59 (95% confidence interval, 0.37–0.86). The Diagnostic Odds Ratio was 2.08 (95% CI, 1.15–6.82). The area under the curve was 0.66 (95% CI, 0.62–0.70)(Fig 4C).

### Dengue Shock Syndrome

None of the included studies reported data comparing TT and ELISA for patients with dengue shock syndrome.

### Subgroup Analysis for Dengue vs ELISA

We conducted a subgroup analysis for the included studies considering only children and adolescents aged 6 months to 15 years. No analysis with adults were conducted, since all 16 included studies did not explore only adults’ participants, when they analyzed adults they mixed the data with children and adolescents

**Fig 7:**
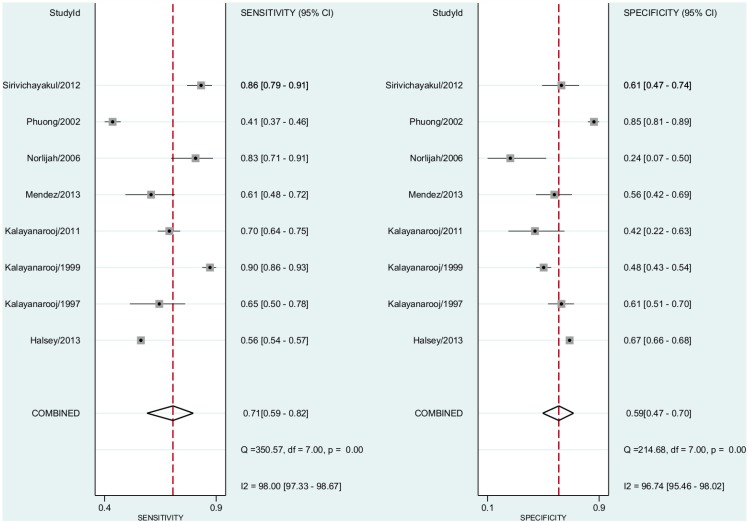
Forest plot for individual studies and pooled sensitivity and specificity for Dengue x ELISA in children and adolescents aged from 6 months to 15 years. Q: chi-squared statistic; df: degrees of freedom; I2: inconsistency of studies’ results; Dashed line means the mean number found across studies.

In this subgroup analysis, we included eight studies including both non-severe dengue fever and DHF cases. The pooled sensitivity for dengue diagnosis was 0.71 (95% CI, 0.59–0.82) and the specificity was 0.59 (95% CI, 0.47–0.70) (Fig 7). The positive predictive value was 1.66 (95% CI, 1.45–1.91). The negative predictive value was 0.52 (95% CI, 0.43–0.64). The Diagnostic Odds Ratio was 3.44 (95% CI, 2.25–5.25). The area under the curve was 0.69 (95% CI, 0.65–0.73).

### Sensitivity Analysis

We conducted the following sensitivity analyses of the Dengue vs ELISA analysis: 1. In order to analysis the impact of the mix of cut-off points reported by studies (≥10 petechiae per one square inch and ≥20 petechiae per one square inch) we repeated the analysis in just studies using the criteria of ≥20 petechiae per one square inch. 2. We conducted another sensitivity analysis removing all studies with high risk of selection bias.

**Fig 8:**
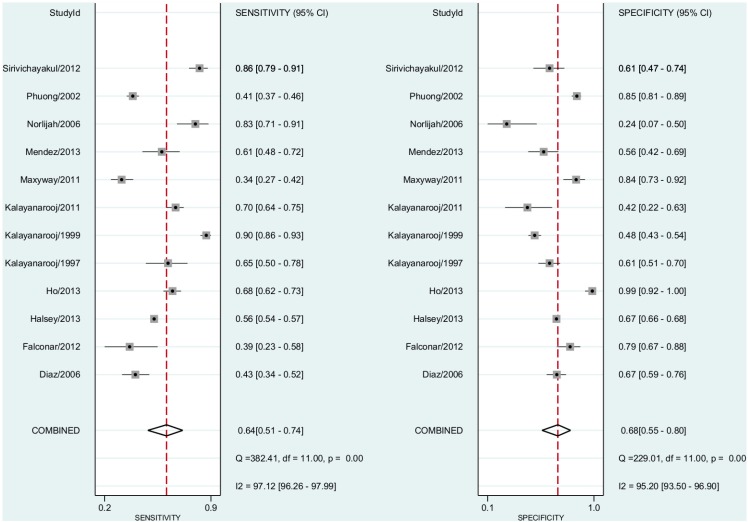
Forest plot for individual studies and pooled sensitivity and specificity for Dengue x ELISA cut-off points reported ≥20 petechiae per one square inch. Q: chi-squared statistic; df: degrees of freedom; I2: inconsistency of studies’ results; Dashed line means the mean number found across studies.

Thus, we included 12 studies including both non-severe dengue fever and DHF cases. The pooled sensitivity for dengue diagnosis was 0.64 (95% CI, 0.51–0.74) and the specificity was 0.68 (95% CI, 0.55–0.80) (Fig 8). The positive predictive value was 1.68 (95% CI, 1.46–1.93). The negative predictive value was 0.56 (95% CI, 0.47–0.66). The Diagnostic Odds Ratio was 3.37 (95% CI, 2.33–4.86). The area under the curve was 0.71 (95% CI, 0.67–0.75).

**Fig 9:**
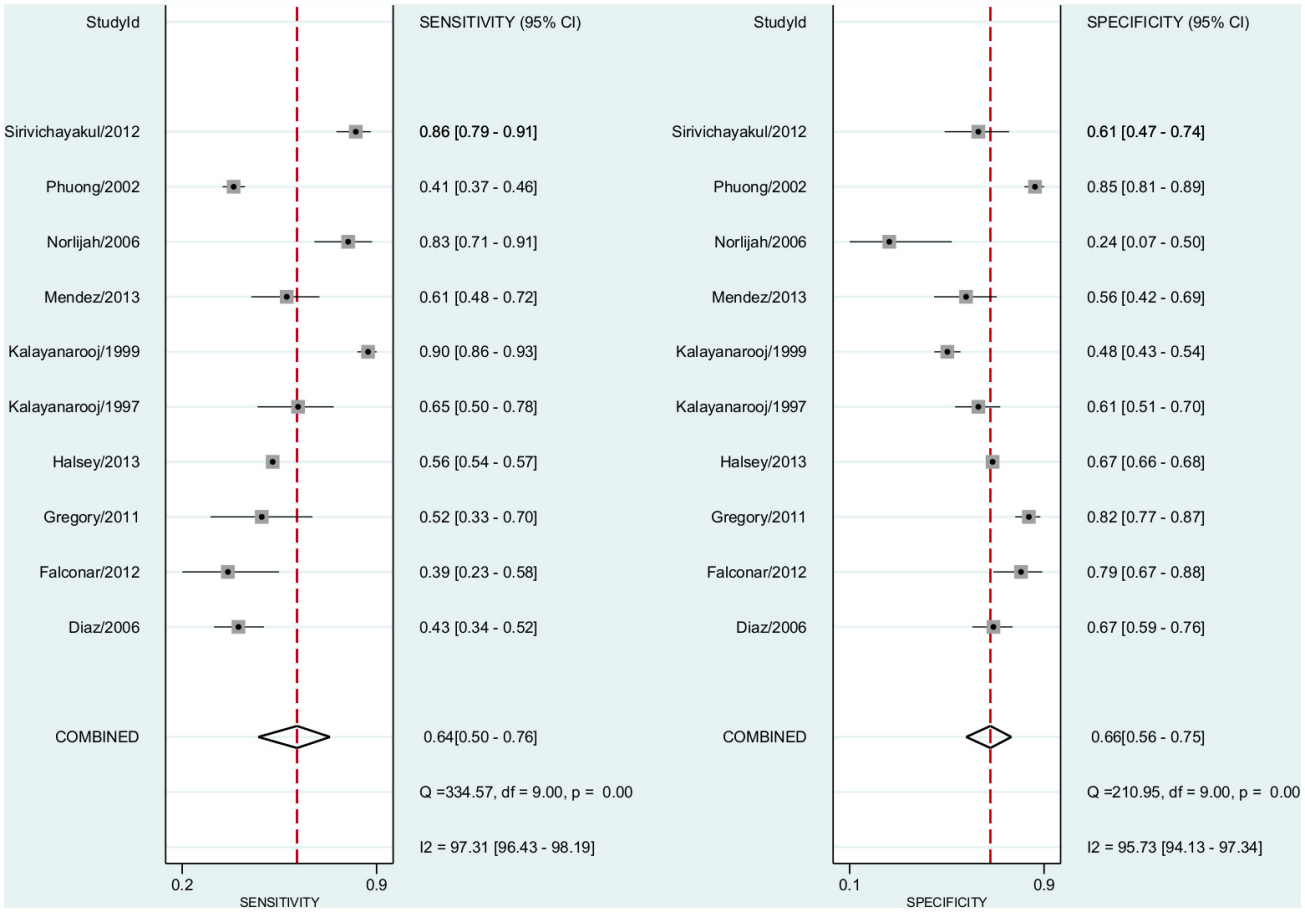
Forest plot for individual studies and pooled sensitivity and specificity for Dengue x ELISA removing six studies with high risk for selection bias. Q: chi-squared statistic; df: degrees of freedom; I2: inconsistency of studies’ results; Dashed line means the mean number found across studies.

We removed six studies with high risk for selection bias. Thus, 10 studies including both non-severe dengue fever and DHF cases were combined. The pooled sensitivity for dengue diagnosis was 0.64 (95% CI, 0.50–0.76) and the specificity was 0.66 (95% CI, 0.56–0.75) (Fig 9). The positive predictive value was 1.74 (95% CI, 1.52–1.98). The negative predictive value was 0.57 (95% CI, 0.48–0.69). The Diagnostic Odds Ratio was 3.37 (95% CI, 2.35–4.85). The area under the curve was 0.70 (95% CI, 0.66–0.74).

### Investigations of Publication Bias and Heterogeneity

**Fig 10:**
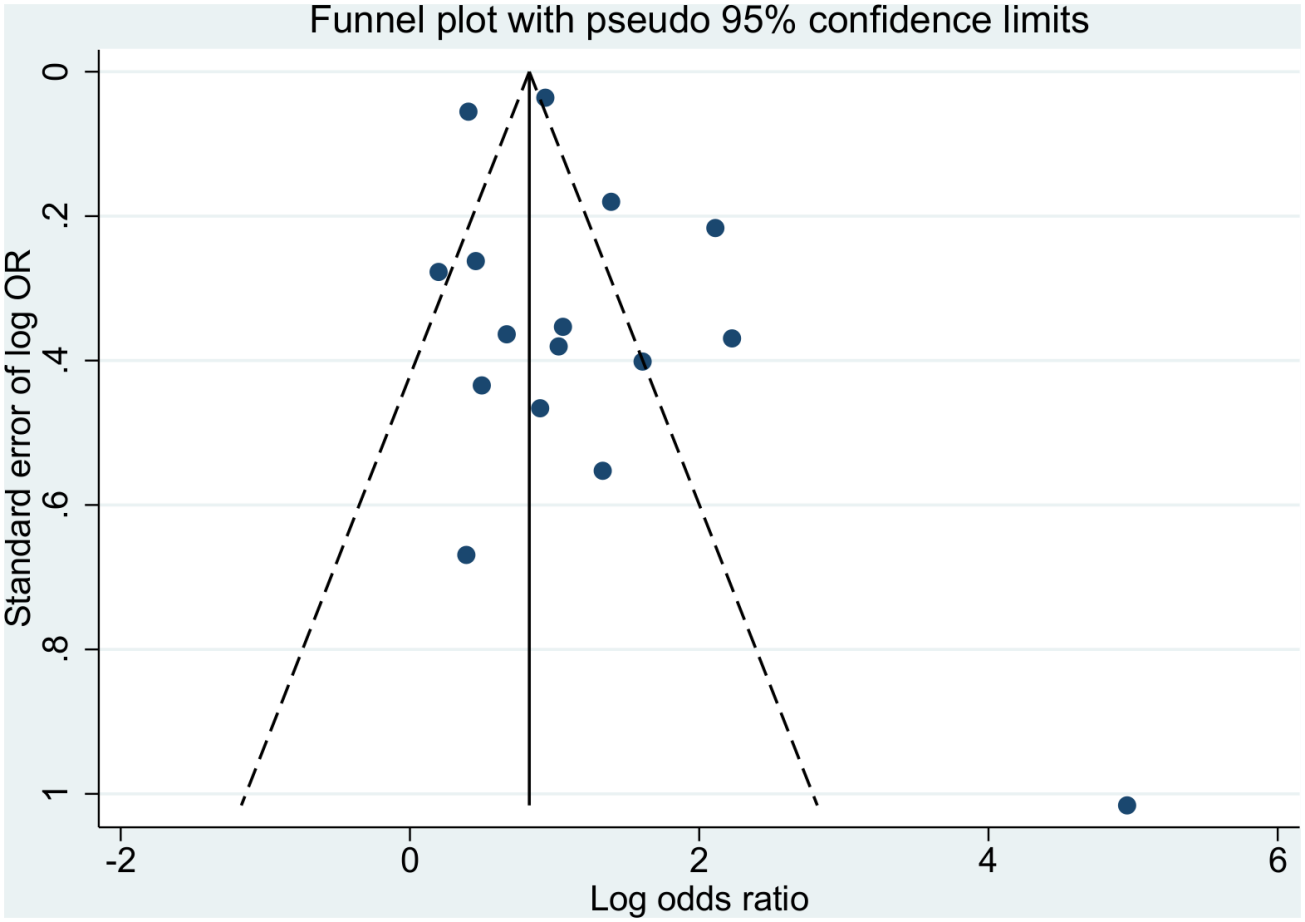
Publication bias presentation using funnel plot for Dengue x ELISA.

Funnel plot asymmetry test revealed evidence of publication bias (Fig 10). The I^2^ statistics were, as expected in diagnostic meta-analyses, over 95% in all three comparisons made (Figs 3, 5 and 6)[26].

## Discussion

Dengue fever is an infection with significant global public health importance. Increasing urbanization and crowding in endemic areas, coupled with failing vector control programs have resulted in a significant increase in cases and major outbreaks since the 1950’s[11]. This is the first systematic review and meta-analysis to specifically investigate and compare the utility of the tourniquet test to diagnose dengue infection compared to the widely-used standard laboratory ELISA testing.

Overall, our results demonstrate that the TT is a relatively poor diagnostic test for dengue of any severity. When assessed by ROC analysis, low AUCs (≤0.70) suggest that the TT, as an isolated diagnostic test and in comparison to ELISA, should be classified as a “relatively poor” test.[103]

Funnel plot analysis suggests that there may be a major element of publication bias in the previous reporting of tourniquet test usefulness. Many studies were observed to have overly extreme results, i.e. have a large effect for positive TT and large effect against TT. Reasons for this may include small sample size with wide standard errors, or other problems in study design, with resulting overemphasis in reporting positive or confirmatory results. Resulting estimates of TT efficacy may there have demonstrated a significant skew towards overly positive effects.

In our subgroups analysis, we removed the studies that mixed adults and kept only children and adolescents from 6 months to 15 years of age. We did not find any significant change in utility of TT in diagnosing dengue infection. We did further sensitivity analyses considering both a diagnostic cut-off of ≥20 petechiae per one square inch and a repeat analysis after removing studies at high risk of bias for patient selection. Neither of these analyses led to any significant difference in our findings. This is of interest, as using a higher, stricter cutoff would generally reduce sensitivity but increase specificity for diagnosis. Lack of an effect seen when increasing the threshold to ≥20 petechiae here, suggests limited biological correlation of this clinical observation. Additional analyses demonstrated that data from individual studies were scattered and included a wide range of participant age-groups and geographic regions. Ideally, we would also have liked to investigate the performance of the TT in different age-groups using a range of cutoff thresholds, for example, ≥10 petechiae in children, ≥20 petechiae in adults. It was not possible to extract these age-group specific data from many of the primary studies however.

Considering the subgroup analysis, we could hypothesize that increasing the threshold for diagnosing dengue the sensitivity would decrease and specificity increase, however this hypothesis was not confirmed, the lack effect on the outcomes shows the need to use a more rigorous test to diagnose dengue.

Using the GRADE approach to assess the quality of the evidence generated in this study, we can classify it as low, which means that “*further research is very likely to have an important impact on our confidence in the estimate of effect and is likely to change the estimate or any estimate of effect is very uncertain*”, the evidence was downgraded due to imprecision (wide confidence intervals) and inconsistency (widely different estimates) across the included studies.

Further limitations to our analysis of data currently available in the literature may arise from heterogeneity in the time periods at which the tests were performed, or the number of occasions on which the test was repeated prior to getting a positive result. Additional practical reasons for previous overestimation of the efficacy of the TT may include difficulties in interpreting a positive result in individuals with different skin pigmentation or variation in the virulence or pathogenicity of strains resulting in higher rates of capillary permeability, for example in South East Asian genotypes of the DEN-2/3 serotypes.

Here, we have assessed and presented the best available evidence for use of the TT in making a diagnosis of dengue infection. Clearly the TT should not be used in isolation for making a diagnosis of dengue, however given the evidence available it is doubtful as to whether the test offers any additional benefit over and above careful clinical evaluation. Inconsistencies in data reporting in the primary study datasets also render assessment of whether the TT maybe useful for particular population/disease subgroups currently infeasible.

### Conclusions

The clinical diagnosis of dengue is challenging as disease presentation is almost indistinguishable to many other infections commonly found in the tropics[104]. Current WHO recommendations suggest a combination of clinical history, leukopenia and the tourniquet test result to make a diagnosis if ELISA testing is not available or prior to the availability of results. Given the requirement for paired sera samples in many areas where dengue is endemic to demonstrate an increase in antibody titre, reliance on clinical diagnosis will be still greater.

While still widely used, our analyses suggest that data supporting routine use of the tourniquet test is, at best, relatively poor, however it is important to consider that the quality of the evidence is low due to imprecision and inconsistency across the included studies. Furthermore, the data used to underpin current international recommendations likely overestimate its utility. Over reliance on the use of the TT to support a clinical diagnosis of dengue infection may result in misdiagnosis of patients and inaccurate estimates of disease incidence; relatively low sensitivity but higher specificity suggest that disease incidence may be underestimated if the TT is overly relied on. While current recommendations should be re-examined in light of these findings, replacement of the tourniquet test in routine clinical practice will only come once improved point-of-care diagnostics are made more widely available, especially in resource-poor areas.

## References

[1] Deen JL, Harris E, Wills B, Balmaseda A, Hammond SN, et al (2006) The WHO dengue classification and case definitions: time for a reassessment. Lancet 368: 170–173. 16829301 10.1016/S0140-6736(06)69006-5

[2] Morens DM, Fauci AS (2008) Dengue and hemorrhagic fever: a potential threat to public health in the United States. Jama 299: 214–216. 10.1001/jama.2007.31-a 18182605

[3] Kularatne SA (2015) Dengue fever. Bmj 351: h4661 10.1136/bmj.h4661 26374064

[4] Kabra S, Jain Y, Pandey R, Madhulika, Singhal T, et al (1999) Dengue haemorrhagic fever in children in the 1996 Delhi epidemic. Transactions of the Royal Society of Tropical Medicine and Hygiene 93: 294–298. 10492762 10.1016/s0035-9203(99)90027-5

[5] Manjith N, Aravind MA, Thilothammal N, Prema R, Sargunam CSR, et al (2002) Dengue fever epidemic in Chennai—a study of clinical profile and outcome. Indian Pediatrics 39: 1027–1033. 12466573

[6] Mendez A, Gonzalez G (2003) Dengue haemorrhagic fever in children: ten years of clinical experience. [Spanish]. Biomedica: revista del Instituto Nacional de Salud 23: 180–193. 12872557

[7] Muhammad A, Khazindar A, Lubbad E, Shahid B, Alfi A, et al (2006) Characteristics of Dengue Fever in a large public hospital, Jeddah, Saudi Arabia. Journal of Ayub Medical College 18: 9–13. 16977805

[8] Munir M, Husada T, Mustadjab I (1982) Dengue haemorrhagic fever. A problem of clinical diagnosis and proposal for using a scoring system. Paediatrica Indonesiana 22: 11–22. 7177673

[9] Islam R, Salahuddin M, Ayubi MS, Hossain T, Majumder A, et al (2015) Dengue epidemiology and pathogenesis: images of the future viewed through a mirror of the past. Virol Sin. 10.1007/s12250-015-3624-1 PMC8200867 26494479

[10] Prevention CfDCa (2015) Dengue Homepage.

[11] (WHO) WHO (2011) Comprehensive Guidelines for Prevention and Control of Dengue and Dengue Haemorrhagic Fever. 1st ed Geneva: WHO.

[12] (WHO). WHO (2009) Dengue: Guidelines for Diagnosis, Treatment, Prevention and Control.: Geneva: WHO; 2009. 23762963

[13] Antunes A, Oliveira G, Nunes L, Guedes Filho L, Prado R, et al (2013) Evaluation of the diagnostic value of the tourniquet test in predicting severe dengue cases in a population from Belo Horizonte, State of Minas Gerais, Brazil. 46: 542–546. 10.1590/0037-8682-0161-2013 24270244

[14] Falconar A, Romero-Vivas C (2012) Simple Prognostic Criteria can Definitively Identify Patients who Develop Severe Versus Non-Severe Dengue Disease, or Have Other Febrile Illnesses. Journal of Clinical Medicine Research 4: 33–44. 10.4021/jocmr694w 22383925 PMC3279499

[15] Diaz FA, Martinez RA, Villar LA (2006) Clinical criteria to diagnose dengue in its early stages. [Spanish]. Biomedica: revista del Instituto Nacional de Salud 26: 22–30. 16929900

[16] Halsey E, Vilcarromero S, Forshey B, Rocha C, Bazan I, et al (2013) Performance of the tourniquet test for diagnosing dengue in Peru. American Journal of Tropical Medicine & Hygiene 89: 99–104. 23716410 10.4269/ajtmh.13-0103 PMC3748494

[17] Ho TS, Wang SM, Lin YS, Liu CC (2013) Clinical and laboratory predictive markers for acute dengue infection. Journal of biomedical science 20: 75 10.1186/1423-0127-20-75 24138072 PMC4015130

[18] Hoang LP, Vries P, de, Nga T, Giao P, et al (2006) Dengue as a cause of acute undifferentiated fever in Vietnam. BMC Infectious Diseases 6. 10.1186/1471-2334-6-123 PMC1579223 16869969

[19] Kalayanarooj S, Nimmannitya S, Suntayakorn S, Vaughn D, Nisalak A, et al (1999) Can doctors make an accurate diagnosis of dengue infections at an early stage? Dengue Bulletin 23: 1–9.

[20] Kalayanarooj S, Vaughn D, Nimmannitya S, Green S, Suntayakorn S, et al (1997) Early clinical and laboratory indicators of acute dengue illness. Journal of Infectious Diseases 176: 313–321. 9237695 10.1086/514047

[21] Sawasdivorn S, Vibulvattanakit S, Sasavatpakdee M, Iamsirithavorn S (2001) Efficacy of clinical diagnosis of dengue fever in paediatric age groups as determined by WHO case definition 1997 in Thailand. Dengue Bulletin 25: 56–64.

[22] Peeling RW, Artsob H, Pelegrino JL, Buchy P, Cardosa MJ, et al Evaluation of diagnostic tests: dengue. Nat Rev Micro. 10.1038/nrmicro2459 21548185

[23] Whiting PF, Rutjes AW, Westwood ME, Mallett S, Deeks JJ, et al (2011) QUADAS-2: a revised tool for the quality assessment of diagnostic accuracy studies. Annals of internal medicine 155: 529–536. 10.7326/0003-4819-155-8-201110180-00009 22007046

[24] Deeks JJ (2001) Systematic reviews of evaluations of diagnostic and screening tests Systematic Reviews in Health Care: Meta-Analysis in Context, Second Edition: 248–282. 10.1136/bmj.323.7305.157 PMC1120791 11463691

[25] Deeks JJ, Higgins J, Altman DG (2008) Analysing Data and Undertaking Meta‐Analyses. Cochrane Handbook for Systematic Reviews of Interventions: Cochrane Book Series: 243–296.

[26] Higgins JP, Green S (2008) Cochrane handbook for systematic reviews of interventions: Wiley Online Library.

[27] Ahmed FU, Mahmood CB, Sharma JD, Hoque SM, Zaman R, et al (2001) Dengue and dengue haemorrhagic fever in children during the 2000 outbreak in Chittagong, Bangladesh. Dengue Bulletin 25: 33–39.

[28] Arif KM, Mohammed FR, Nur Z, Shams Md Z, Alam Md B, et al (2009) Clinical profile and outcome of dengue hemorrhagic fever in a Tertiary Care Hospital in Dhaka. Journal of Medicine 10: 12–15.

[29] Awasthi S, Singh VK, Kumar S, Kumar A, Dutta S (2012) The changing clinical spectrum of Dengue fever in the 2009 epidemic in north India: A tertiary teaching hospital based study. Journal of Clinical and Diagnostic Research 6: 999–1002.

[30] Ayyub M, Khazindar A, Lubbad E, Barlas S, Alfi A, et al (2006) Characteristics of dengue fever in a large public hospital, Jeddah, Saudi Arabia. Journal of Ayub Medical College, Abbottabad: JAMC 18: 9–13. 16977805

[31] Bandyopadhyay S, Lum LCS, Kroeger A (2006) Classifying dengue: A review of the difficulties in using the WHO case classification for dengue haemorrhagic fever. Tropical Medicine and International Health 11: 1238–1255. 16903887 10.1111/j.1365-3156.2006.01678.x

[32] Cavalcanti LC; Vilar D; Holanda S; da, Escossia K; Souza-Santos R (2010) Clinical and epidemiological characterization of dengue hemorrhagic fever cases in northeastern, Brazil. [Portuguese]. Revista da Sociedade Brasileira de Medicina Tropical 43: 355–358. 20802929 10.1590/s0037-86822010000400003

[33] Chairulfatah A, Setiabudi D, Ridad A, Colebunders R (1995) Clinical manifestations of dengue haemorrhagic fever in children in Bandung, Indonesia. Annales de la Societe Belge de Medecine Tropicale 75: 291–295. 8669976

[34] Chuansumrit A, Tangnararachakit K, Sirachainan N, Khositseth A, Kuptanon T, et al (2010) Dengue hemorrhagic fever in hemophilic patients: Aggravation of bleeding risk. Haematologica 95: 72. 10.1111/j.1365-2516.2010.02413.x 21070495

[35] Chutinimitkul S, Payungporn S, Theamboonlers A, Poovorawan Y (2005) Dengue typing assay based on real-time PCR using SYBR Green I. Journal of Virological Methods 129: 8–15. 15941596 10.1016/j.jviromet.2005.05.006

[36] Daniel R, Rajamohanan, Philip AZ (2005) A study of clinical profile of dengue fever in Kollam, Kerala, India. Dengue Bulletin 29: 197–202.

[37] Diaz-Quijano FA, Martinez-Vega RA, Villar-Centeno LA (2008) Early predictors of haemorrhage in acute febrile syndrome patients from Bucaramanga, Colombia: a dengue endemic area. Singapore Medical Journal 49: 480–482. 18581022

[38] Diaz-Quijano FA, Martinez-Vega RA, Villar-Centeno LA (2009) Correlation between variation of hematocrit and other indicators of severity in dengue. [Spanish]. Colombia Medica 40: 408–414.

[39] Diaz-Quijano FA, Villar-Centeno LA, Martinez-Vega RA (2010) Predictors of spontaneous bleeding in patients with acute febrile syndrome from a dengue endemic area. Journal of Clinical Virology 49: 11–15. 10.1016/j.jcv.2010.06.011 20663710

[40] Eram S, Setyabudi Y, Sadono T (1979) Epidemic dengue hemorrhagic fever in rural Indonesia. II. Clinical studies. 28: 711–716. 464192

[41] Faridi M, Anju A, Manish K, Abedin S (2008) Clinical and biochemical profile of dengue haemorrhagic fever in children in Delhi. Tropical Doctor 38: 28–30. 10.1258/td.2007.006158 18302860

[42] Filho F, Mota M, Araujo E, Souza N, Fontes R, et al (2012) Evaluation of the tourniquet test for the diagnosis of dengue infection. 89: 319–320.

[43] Fonseca BA, Castro-Jorge LA, Sobral MCM, Esposito DL, Feitosa ALP, et al (2012) Evaluation of the performance of clinical and laboratorial dengue diagnosis during an epidemic in a medium-sized city in southeast brazil. American Journal of Tropical Medicine and Hygiene 1): 334.

[44] Gomber S, Ramachandran VG, Kumar S, Agarwal KN, Gupta P, et al (2001) Hematological observations as diagnostic markers in dengue hemorrhagic fever—a reappraisal. Indian pediatrics 38: 477–481. 11359973

[45] Gregory C, Alvarado-Domenech L, Colon L, Cruz-Rivera R, Cuyar-Bermudez L, et al (2010) Combined utility of tourniquet test and white blood cell count as triage criteria for dengue in the Americas. American Journal of Tropical Medicine and Hygiene 1): 148–149.

[46] Guzman M, Vazquez S, Martinez E, Alvarez M, Rodriguez R, et al (1996) Dengue in Nicaragua, 1994: reintroduction of serotype 3 in the Americas. [Spanish]. Boletin de la Oficina Sanitaria Panamericana 121: 102–110. 8983243

[47] Hanif M, Sarkar DN, Amin MR, Basher A, Ahmed T (2011) Clinical profile and outcome of patients with dengue syndrome in hospital care. Journal of Medicine 12: 131–138.

[48] Horstick O, Farrar J, Lum L, Martinez E, San Marti JL, et al (2012) Reviewing the development, evidence base, and application of the revised dengue case classification. Pathogens and Global Health 106: 94–101. 10.1179/2047773212Y.0000000017 22943544 PMC3408880

[49] Horstick O, Jaenisch T, Martinez E, Kroeger A, See L, et al (2014) Comparing the usefulness of the 1997 and 2009 WHO dengue case classification: a systematic literature review. [Review]. American Journal of Tropical Medicine & Hygiene 91: 621–634. 24957540 10.4269/ajtmh.13-0676 PMC4155569

[50] Ismail N, Kampan N, Mahdy Z, Jamil M, Razi Z (2006) Dengue in pregnancy. Southeast Asian Journal of Tropical Medicine & Public Health 37: 681–683. 17121293

[51] Jhamb R, Kumar A, Ranga GS, Rathi N (2010) Unusual manifestations in Dengue outbreak 2009, Delhi, India. Journal of Communicable Diseases 42: 255–261. 22471194

[52] Kalayanarooj S, Nimmannitya S (2003) Clinical Presentations of Dengue Hemorrhagic Fever in Infants Compared to Children. Journal of the Medical Association of Thailand 86: S673–S680. 14700166

[53] Kapse AS (2003) Dengue illness: Approach to clinical diagnosis and management. Indian Journal of Practical Pediatrics 5: 213–221.

[54] Kittitrakul C, Silachamroon U, Phumratanaprapin W, Krudsood S, Wilairatana P, et al (2015) Liver function tests abnormality and clinical severity of dengue infection in adult patients. Journal of the Medical Association of Thailand 98. 25764606

[55] Kulkarni MJ, Sarathi V, Bhalla V, Shivpuri D, Acharya U (2010) Clinico-epidemiological profile of children hospitalized with dengue. Indian Journal of Pediatrics 77: 1103–1107. 10.1007/s12098-010-0202-2 20890686

[56] Mamani E, Figueroa D, Garcia MP, Garaycochea MC, Pozo EJ (2010) Concurrent infections by two dengue virus serotypes during an outbreak in northwestern peru, 2008. [Spanish]. Revista Peruana de Medicina de Experimental y Salud Publica 27: 16–21. 10.1590/s1726-46342010000100004 21072445

[57] Meltzer E, Heyman Z, Bin H, Schwartz E (2012) Capillary leakage in travelers with dengue infection: implications for pathogenesis. American Journal of Tropical Medicine and Hygiene 86: 536–539. 10.4269/ajtmh.2012.10-0670 22403332 PMC3284377

[58] Mishra B, Gupta PK, Dhiman V, Pujhari SK, Sharma M, et al (2014) Clinical applicability of various dengue diagnostic tests in resource-limited endemic settings. Journal of Global Infectious Diseases 6: 109–113. 10.4103/0974-777X.138504 25191051 PMC4147419

[59] Mourao M, Lacerda M, Macedo V, Santos J (2007) Thrombocytopenia in patients with dengue virus infection in the Brazilian Amazon. Platelets 18: 605–612. 18041652 10.1080/09537100701426604

[60] Munir MA, Alam SE, Khan ZU, Quaid S, Ambreen A, et al (2014) Dengue fever in patients admitted in tertiary care hospitals in Pakistan. JPMA, Journal of the Pakistan Medical Association 64: 553–559. 25272543

[61] Namvongsa V, Sirivichayakul C, Songsithichok S, Chanthavanich P, Chokejindachai W, et al (2013) Differences in clinical features between children and adults with dengue hemorrhagic fever/dengue shock syndrome. Southeast Asian Journal of Tropical Medicine & Public Health 44: 772–779. 24437312

[62] Narayanan M, Aravind M, Ambikapathy P, Prema R, Jeyapaul M (2003) Dengue fever—clinical and laboratory parameters associated with complications. Dengue Bulletin 27: 108–115.

[63] Naskar A, Ghosh M, Mallik S, Pal S, Bandyopadhyay B, et al (2014) A profile of dengue outbreak in adults of an eastern state of India. International Journal of Infectious Diseases 21: 442.

[64] Nunes-Araujo F, Ferreira M, Nishioka S, de A (2003) Dengue fever in Brazilian adults and children: assessment of clinical findings and their validity for diagnosis. Annals of Tropical Medicine and Parasitology 97: 415–419. 12831527 10.1179/000349803235002263

[65] Padbidri V, Adhikari P, Thakare J, Ilkal M, Joshi G, et al (1995) The 1993 epidemic of dengue fever in Mangalore, Karnataka State, India. Southeast Asian Journal of Tropical Medicine and Public Health 26: 699–704. 9139379

[66] Pancharoen C, Thisyakorn U (2001) Dengue virus infection during infancy. Transactions of the Royal Society of Tropical Medicine and Hygiene 95: 307–308. 11491005 10.1016/s0035-9203(01)90245-7

[67] Pandey B, Morita K, Khanal S, Takasaki T, Miyazaki I, et al (2008) Dengue virus, Nepal. Emerging Infectious Diseases 14: 514–515. 10.3201/eid1403.070473 18325280 PMC2570825

[68] Phuong HL, Vries PJd, Nagelkerke N, Giao PT, Hung LQ, et al (2006) Acute undifferentiated fever in Binh Thuan province, Vietnam: imprecise clinical diagnosis and irrational pharmaco-therapy. Tropical Medicine and International Health 11: 869–879. 16772009 10.1111/j.1365-3156.2006.01636.x

[69] Prathyusha CV, Rao MS, Sudarsini P, Rao KUM (2013) Clinico-haematological profile and outcome of dengue fever in children. International Journal of Current Microbiology and Applied Sciences 2: 338–346.

[70] Quiroz-Moreno R, Mendez GF, Ovando-Rivera KM (2006) Clinical utility of ultrasound in the identification of dengue hemorrhagic fever. Revista Medica del Instituto Mexicano del Seguro Social 44: 243–248. 16870119

[71] Ramirez-Ronda CH (1987) Dengue in Puerto Rico: clinical manifestations and management from 1960's to 1987. Puerto Rico health sciences journal 6: 113–118. 3313491

[72] Ramirez-Zepeda M, Velasco-Mondragon H, Ramos C, Penuelas J, Maradiaga-Cecena M, et al (2009) Clinical and epidemiologic characteristics of dengue cases: the experience of a general hospital in Culiacan, Sinaloa, Mexico. [Spanish]. Revista Panamericana de Salud Publica/Pan American Journal of Public Health 25: 16–23. 19341519 10.1590/s1020-49892009000100003

[73] Rao SM, Taggu A, Mutkule D (2013) Dengue fever-an observational studybedside markers. Indian Journal of Critical Care Medicine 17: 25.

[74] Restrepo BN, Piedrahita LD, Agudelo IY, Parra-Henao G, Osorio JE (2012) Frequency and clinical features of dengue infection in a schoolchildren cohort from Medellin, Colombia. Journal of Tropical Medicine 120496. 10.1155/2012/120496 PMC3530854 23304167

[75] Ribeiro VA, R; Resende M; Pavan M; Hoehne E; Souza V; Souza C; Souza M; Wonhrathi M; Cadogan S; Aoki F. (2010) Dengue fever in a Southeastern region of Brazil. Ten years period (1997–2007) clinical and epidemiological retrospective study. International Journal of Infectious Diseases Conference: 14th International Congress on Infectious Diseases 14.

[76] Richards AL, Bagus R, Baso SM, Follows GA, Tan R, et al (1997) The first reported outbreak of dengue hemorrhagic fever in Irian Jaya, Indonesia. American Journal of Tropical Medicine and Hygiene 57: 49–55. 9242317 10.4269/ajtmh.1997.57.49

[77] Rigau-Perez J (1997) Clinical manifestations of dengue hemorrhagic fever in Puerto Rico, 1990–1991. [Spanish]. Revista Panamericana de Salud Publica/Pan American Journal of Public Health 1: 435–443. 10.1590/s1020-49891997000500007 9180059

[78] Setiati TE, Mairuhu ATA, Koraka P, Supriatna M, Mac Gillavry MR, et al (2007) Dengue disease severity in Indonesian children: An evaluation of the World Health Organization classification system. BMC Infectious Diseases 7. 10.1186/1471-2334-7-22 PMC1847434 17386105

[79] Shah GS, Islam S, Das BK (2006) Clinical and laboratory profile of dengue infection in children. Kathmandu University Medical Journal 4: 40–43. 18603866

[80] Sharmin R, Tabassum S, Mamun K, Nessa A, Jahan M (2013) Dengue infection in Dhaka City, Bangladesh. Mymensingh Medical Journal: MMJ 22: 781–786. 24292312

[81] Shibani B, Lum LCS, Kroeger A (2006) Classifying dengue: a review of the difficulties in using the WHO case classification for dengue haemorrhagic fever. Tropical Medicine and International Health 11: 1238–1255. 16903887 10.1111/j.1365-3156.2006.01678.x

[82] Sri Rejeki H, Purwanto SH, Chatab F (1999) Dengue shock syndrome: clinical manifestations, management and outcome—a hospital-based study in Jakarta, Indonesia. Dengue Bulletin 23: 105–106.

[83] Sunil G, Ramachandran VG, Satish K, Agarwal KN, Gupta P, et al (2001) Hematological observations as diagnostic markers in dengue hemorrhagic fever—a reappraisal. Indian Pediatrics 38: 477–481. 11359973

[84] Tham VD, Hien HH, Long HD (1996) Value of the tourniquet sign in the diagnosis of hemorrhagic dengue. [French]. Medecine tropicale: revue du Corps de sante colonial 56: 99. 8767803

[85] Tomashek KM, Garcia-Gubern C, Lorenzi OD, Galarza IE, Acosta H, et al (2010) Acute febrile illness surveillance in a tertiary hospital emergency department: Comparison of influenza and dengue infections. American Journal of Tropical Medicine and Hygiene 1): 139–140. 10.4269/ajtmh.12-0373 PMC3592528 23382160

[86] Uddin MN, Hossain MM, Dastider R, Hasan Z, Ahmed Z, et al (2014) Clinico-pathological profile of dengue syndrome: an experience in a tertiary care hospital, Dhaka, Bangladesh. Mymensingh medical journal: MMJ 23: 774–780. 25481600

[87] Vargas Caballero ME, Aguirre Portuondo TM, Palacios Serrano H (2001) Clinical features of dengue fever in children during the outbreak in Santiago de Cuba. [Spanish]. Revista cubana de medicina tropical 53: 20–23. 11826532

[88] Villar LA, Parra B, Salgado D, Florez J, Bosch I (2011) Clinical implications of adherence to who guidelines for the management of the febrile phase of dengue. American Journal of Tropical Medicine and Hygiene 1): 389.

[89] Wali JP, Biswas A, Aggarwal P, Wig N, Handa R (1999) Validity of tourniquet test in dengue haemorrhagic fever. The Journal of the Association of Physicians of India 47: 203–204. 10999092

[90] Whitehorn J, Farrar J (2010) Dengue. [Review]. British Medical Bulletin 95: 161–173. 10.1093/bmb/ldq019 20616106

[91] Wiwanitkit V (2005) The tourniquet test is still a good screening tool for dengue illness. Tropical Doctor 35: 127–128. 15970060 10.1258/0049475054037093

[92] Yadav S, Anupam S, Dhiren G, Sharma S, Gaurav K (2008) Control of massive bleeding in dengue hemorrhagic fever with severe thrombocytopenia by use of intravenous anti-D globulin. Pediatric Blood & Cancer 51: 812–813. 18683235 10.1002/pbc.21708

[93] Yewale VN (2009) Rational investigation practices in the diagnosis of dengue. Indian Journal of Practical Pediatrics 11: 79–89.

[94] Zhang F, Tang X, Hu X, Lu Y, Chen Y, et al (2007) A clinical, epidemiological and virological study of a dengue fever outbreak in Guangzhou, China—2002–2006. Dengue Bulletin 31: 10–18.

[95] Zhang FC, Chen YQ, Lu YC, Wang J, Chen WS, et al (2005) Analysis on clinical and epidemiological characteristics of 1032 patients with Dengue fever in Guangzhou. [Chinese]. Zhonghua liu xing bing xue za zhi = Zhonghua liuxingbingxue zazhi 26: 421–423. 16185453

[96] Gregory C, Lorenzi O, Colon L, Garcia A, Santiago L, et al (2011) Utility of the tourniquet test and the white blood cell count to differentiate dengue among acute febrile illnesses in the emergency room. 5. 10.1371/journal.pntd.0001400 PMC3232191 22163057

[97] Kalayanarooj S (2011) Dengue classification: current WHO vs. the newly suggested classification for better clinical application? Journal of the Medical Association of Thailand 94: 74–84. 22043757

[98] Mayxay M, Phetsouvanh R, Moore C, Chansamouth V, Vongsouvath M, et al (2011) Predictive diagnostic value of the tourniquet test for the diagnosis of dengue infection in adults. Tropical Medicine and International Health 16: 127–133. 10.1111/j.1365-3156.2010.02641.x 20958892 PMC3073123

[99] Mendez A, Gonzalez G (2003) [Dengue haemorrhagic fever in children: ten years of clinical experience]. [Spanish]. Biomedica 23: 180–193. 12872557

[100] Norlijah O, Khamisah A, Kamarul A, Paeds M, Mangalam S (2006) Repeated tourniquet testing as a diagnostic tool in dengue infection. Medical Journal of Malaysia 61: 22–27. 16708730

[101] Phuong CXT, Ngo TN, Wills BA, Kneen R, Ha NTT, et al (2002) Evaluation of the World Health Organization standard tourniquet test and a modified tourniquet test in the diagnosis of dengue infection in Viet Nam. Tropical Medicine and International Health 7: 125–132. 11841702 10.1046/j.1365-3156.2002.00841.x

[102] Sirivichayakul C, Limkittikul K, Chanthavanich P, Jiwariyavej V, Chokejindachai W, et al (1520) Dengue infection in children in Ratchaburi, Thailand: a cohort study. II. Clinical manifestations. PLoS Neglected Tropical Diseases [electronic resource] 6. 10.1371/journal.pntd.0001520 PMC3289597 22389735

[103] Metz CE (1978) Basic principles of ROC analysis. Semin Nucl Med 8: 283–298. 112681 10.1016/s0001-2998(78)80014-2

[104] Guzman MG, Alvarez M, Halstead SB (2013) Secondary infection as a risk factor for dengue hemorrhagic fever/dengue shock syndrome: An historical perspective and role of antibody-dependent enhancement of infection. Archives of Virology 158: 1445–1459. 10.1007/s00705-013-1645-3 23471635

